# Oncocytic variant of sialadenoma papilliferum – a rare salivary gland tumor: A case report

**DOI:** 10.4317/jced.59284

**Published:** 2022-07-01

**Authors:** Heidi Tuominen, Aaro Turunen, Jaana Willberg, Hanna Laine

**Affiliations:** 1 DDS, PhD. Department of Oral Pathology and Oral Radiology, Institute of Dentistry, Faculty of Medicine, University of Turku, Turku, Finland; 2 DDS, PhD. Welfare Division, Oral Health Care, City of Turku, Turku, Finland; 3 DDS, PhD. Department of Oral and Maxillofacial Surgery, Institute of Dentistry, Faculty of Medicine, University of Turku, Turku, Finland; 4 DDS, PhD. Department of Oral and Maxillofacial Diseases, Oral and Maxillofacial Surgery, Turku University Hospital, Turku, Finland; 5 DDS, PhD. Department of Pathology, Turku University Hospital, Turku, Finland; 6 DDS, PhD. Department of Oral and Maxillofacial diseases, Clinicum, Faculty of Medicine, University of Helsinki and Helsinki University Hospital, Helsinki, Finland

## Abstract

**Background:**

Sialadenoma papilliferum (SP) is a rare minor salivary gland neoplasm that accounts for less than 1% of all salivary gland tumors. The tumor typically affects older people, presenting most commonly as a slow-growing tumor of the hard palate, although other anatomical subsites, comprising the oral cavity and parotid glands, have also been reported.

**Case Report:**

We report a SP occurring in a 90-year-old female. The patient described feeling a nodule on her palate for several years. The lesion was painless and clinically resembled a round craterlike ulceration of diameter 3 mm. The excisional biopsy was diagnosed histologically as SP. Here, we report the clinicopathological and radiological findings of palatal SP.

**Conclusions:**

SP is a rare, benign salivary gland neoplasm, and there are only a few cases described in the literature. Although mostly benign, malignant transformation can occur and should prompt the clinician to ensure complete removal of the tumor tissue.

** Key words:**Sialadenoma papilliferum, minor salivary gland tumor, histopathology, oral pathology, case report.

## Introduction

Sialadenoma papilliferum (SP) is a rare, benign neoplasm arising from the minor salivary glands of the oral cavity (1,2). It comprises less than 1% of all minor salivary gland tumours (SGTs) (1,3,4). According to the literature, patient age at diagnosis ranges from 2 to 91 years, with a mean age of 59 years (2–5). Both sexes are equally affected (6). The most common location is the hard palate (80%), followed by buccal mucosa, upper lip, retromolar pad, and parotid gland (1–3,7). Lesions usually do not exceed a diameter of 2 cm, often being less than one cm (1,2,7). SP often appears as a white, exophytic, round, asymptomatic, well-circumscribed, slow-growing mass with a papillary surface (2,4,7). The most common differential diagnosis should screen for malignancy, squamous cell papilloma, hemangioma, fibroma, and mucocele (1,2). SP is not related to human papillomavirus (HPV), although the clinical appearance may be similar to squamous cell papilloma (4,7).

The main treatment for SP, as for many other benign SGTs, is surgical excision, and the recurrence rate seems to be low (around 7.4%) (1–3,7,8). Despite SP having an excellent prognosis, some reports of malignant transformation in pre-existing SP have been made but not conclusively proven (1,8,9). Here, we present a case of minor salivary gland SP.

## Case Report

A 90-year-old woman was referred for dental examination in the public Oral Health Care Center of Turku prior to planned arthroplasty and prosthetization of both shoulder joints. The patient, although elderly, was in stable general health and was taking Amitriptyline® for arthritic pain, and Furosemide® and Warfarin® daily for well-controlled cardiac failure and atrial fibrillation. Her teeth had been extracted more than 50 years previously, and her full-arch dentures fit well. Of note, the patient records revealed that a small, reddish lesion was described in the hard palate three years earlier. The lesion was regarded as inflammation of a minor salivary duct opening, and further investigations or follow-up were not arranged.

**Figure 1:**
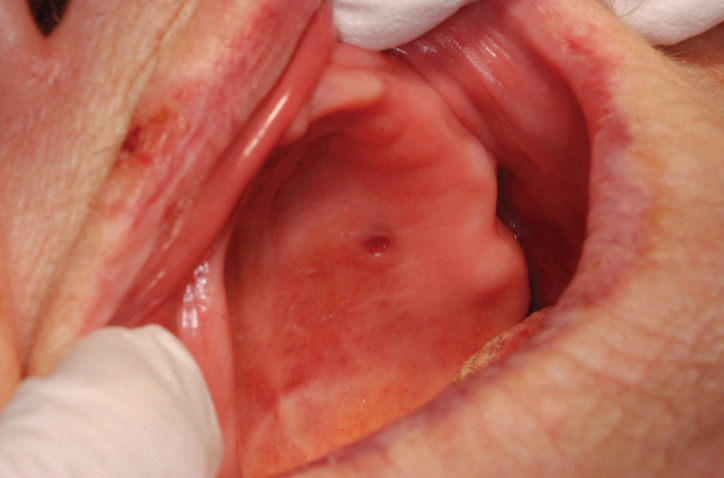
Clinical presentation of the roundish groove-like ulceration (diameter 3 mm) in the hard palate.

Intraoral examination revealed a small, round, reddish ulceration of diameter 3 mm on the left side of the hard palate (Fig. 1). The patient did not remember a previous trauma to the area but recalled that the lesion had been present for several years. Palpation of the lesion did not cause discomfort. For excision of the lesion, the patient was referred to the Department of Oral and Maxillofacial Diseases, Turku University Hospital, Turku, Finland.

**Figure 2:**
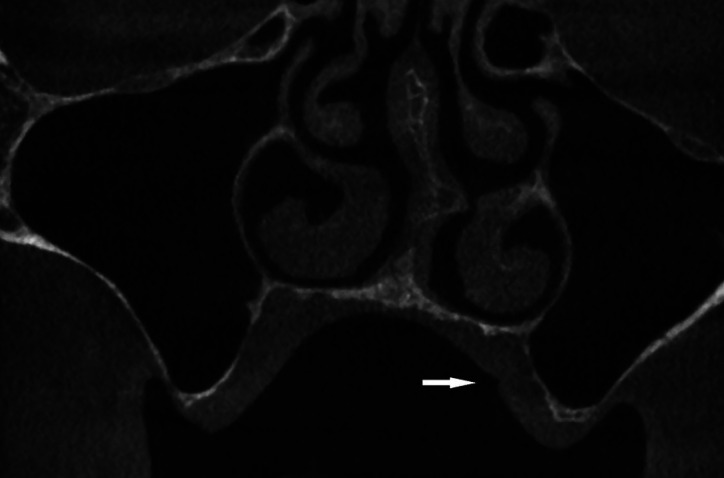
The cone beam computed tomography examination of the lesion area (white arrow). No bony pathology was found.

A cone beam computed tomography (CBCT) examination was performed to rule out bone pathology and retained roots. No pathological changes of the bone underlying the lesion were detected (Fig. 2). A complete blood count and the international normalized ratio (INR) were within the normal range, and the lesion was biopsied using a 4 mm tissue punch. Under the epithelial surface, the lesion resembled granulation tissue.

**Figure 3:**
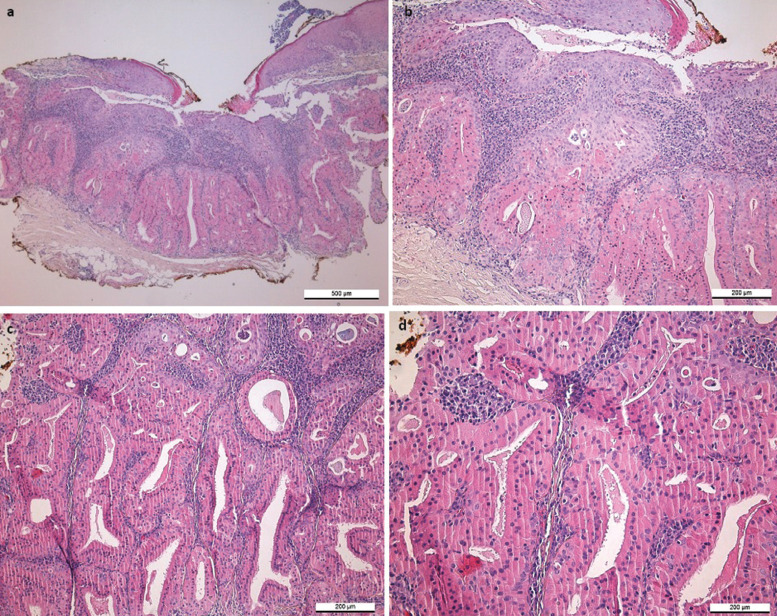
Histopathological findings of the oncocytic variant of sialadenoma papilliferum. Oncocytic cells forming ductal structures in the lamina propria merge with the overlying surface epithelium (a, b). Ductal structures are entirely lined by oncocytic cells with eosinophilic cytoplasm and round nuclei (c, d). Inflammatory infiltrate composed of lymphocytes and plasma cells is seen in the stroma.

Microscopic examination revealed a tumor consisting of oncocytic cells forming ductal structures in the lamina propria (Fig. 3). Oncocytic cells had an eosinophilic cytoplasm and round nuclei. The ductal structures merged with the surface epithelium. In the stroma, there was abundant chronic inflammatory infiltrate composed mainly of plasma cells and lymphocytes. The diagnosis of SP was made based on the histology, and immunohistochemistry was not performed.

## Discussion

SP is a rare, benign SGT, with only a few cases described in the literature. Oncocytic metaplasia has been shown in the histology of SP previously (1), and recently Hsieh *et al*. (5) suggested that SP has two histological variants, namely classic and oncocytic. Our patient case represents an oncocytic type of SP in which the papillary endophytic ductal component composed of oncocytic cells merges with the stratified epithelium above forming papillary structures (Figure 3). Classic SP, in turn, has a papillary squamous surface and an endophytic part of ductal structures that is composed of columnar or cuboidal cells forming bilayered or multilayered structures (5,10). Interestingly, Hsieh *et al*. (5) showed that conventional SP presents with SOX10 expression and BRAF V600E mutations comparably to syringocystadenoma papilliferum of the skin, whereas the oncocytic variant of SP lacks BRAF mutations and does not express SOX10.

Inflammation and sialolithiasis have been suggested as etiologic factors for SP. In our case, the inflamed salivary duct opening noted several years prior to SP diagnosis may have contributed to SP development (6). Importantly, the palate has an abundant quantity of minor salivary glands, offering a common site for different SGTs. Unfortunately, in the oral cavity, malignancies comprise 50% of SGTs (11). In Finland and Denmark, adenoid cystic carcinoma (ACC) is the most common malignant SGT, followed by mucoepidermoid carcinoma (12,13), and in the oral cavity ACC is diagnosed commonly in the palate (14). Bearing this fact in mind, planning the surgical removal of a suspected SGT is important to ensure adequate excision margins. Owing to the generally indolent and benign nature of SP, a local excision usually results in a cure. Nevertheless, malignant transformation in regions of SP has been observed and should prompt the clinician to ensure the complete removal of tumor tissue (8). In our case, no erosion of palatal bone was detected, although lytic bone lesions owing to SP are possible, albeit readily removed by curettage (15). Our patient was scheduled for a local excision of the tumor. Koc and coworkers (15) described the first removal of SP using robotic surgery (TORS), which might facilitate the minimally invasive excision of SP from anatomically difficult areas in the future. Lastly, adequate follow-up to detect possible recurrences plays an important role in managing patients with this rare tumor.
